# Optimizing a Novel eDNA‐Based Framework for Reef Fish Biodiversity Monitoring Using an Autonomous Filtration System and in situ Nanopore Sequencing

**DOI:** 10.1002/ece3.73254

**Published:** 2026-03-23

**Authors:** Lucie Cartairade, Julie Poulain, Quentin Carradec, Sophie Mangenot, Patrick Wincker, Serge Planes

**Affiliations:** ^1^ CNRS‐EPHE‐UPVD, UAR3278 CRIOBE PSL Research University Perpignan France; ^2^ Génomique Métabolique, Genoscope, Institut François Jacob, CEA, CNRS Univ Evry, Université Paris‐Saclay France; ^3^ Research Federation for the Study of Global Ocean Systems Ecology and Evolution, R2022/Tara Oceans GO‐SEE Paris France

**Keywords:** eDNA, fish biodiversity, French Polynesia, metabarcoding, nanopore

## Abstract

Environmental DNA (eDNA) metabarcoding emerged as a powerful method for biodiversity monitoring, offering non‐invasive, reproducible, and scalable assessments. However, its implementation in marine environments and remote regions still presents significant technical and logistical challenges. Here, we tested several methodologies to propose a comprehensive field‐adapted eDNA metabarcoding workflow, from sampling to taxonomic analysis, designed for the specific challenge of fish biodiversity monitoring in coral reefs, but transferable to various aquatic ecosystems. We use large‐volume water sampling using a custom‐built autonomous underwater filtration system, which reduces contamination risks, simplifies logistics, and enhances sampling consistency. In this frame, we assess the efficiency of our custom underwater filtration system, different filter porosities, and water volumes to optimize DNA yield, showing that 20 L filtered on a 1.2 μm pore‐size filter was optimal for collecting fish eDNA. We chose the mitochondrial 12S rRNA for DNA amplification then Oxford Nanopore Technologies sequencing for its portability and compatibility with tropical fieldwork. We developed a high‐resolution 12S reference database encompassing around 400 fish sequences, representing over 50% of the known fish biodiversity in French Polynesia. Finally, we benchmark eDNA‐based biodiversity data against traditional visual census observations across multiple reef habitats and temporal replicates. Our approach enables high‐resolution monitoring both at spatial and temporal scales while providing a more comprehensive assessment of fish biodiversity richness compared to visual census methods. Diel sampling revealed pronounced temporal structure, with nocturnal taxa enriched at night. This study validates a fully integrated eDNA metabarcoding workflow, from field collection to taxonomic analysis, in diverse and remote marine ecosystems, providing a scalable and cost‐efficient framework solution for long‐term biodiversity monitoring in remote regions.

## Graphical abstract

This study presents a comprehensive eDNA metabarcoding protocol optimized for monitoring fish biodiversity in coral reefs and other remote marine environments. By combining an autonomous underwater filtration system, Oxford Nanopore sequencing, and a 12S reference database specifically developed for French Polynesia, the method enables efficient and field‐adapted sampling. This workflow provides high‐resolution spatiotemporal assessments of biodiversity, outperforming traditional visual surveys.

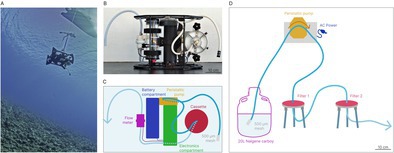

## 1 Introduction

Coral reefs are among the most biodiverse ecosystems on Earth, and as such, they have been designated as a biodiversity hotspot (Hughes et al. 2002; Wagner et al. 2020; Roberts et al. 2002). The health of fish populations within these ecosystems, which serves as a critical indicator of overall reef health, is essential for monitoring the well‐being of these ecosystems (Holbrook et al. 2015). However, assessing the biodiversity of reef fish is particularly challenging, requiring taxonomic expertise, specialized diving skills, and being subject to observer bias. Visual censuses alone, while valuable, are often limited by these factors and lack the sensitivity needed for long‐term and detailed biodiversity assessments. Visual methods may often miss cryptic, rare, elusive, or nocturnal species, are prone to misidentification of morphologically similar taxa, and rely heavily on the observer's taxonomic expertise. In contrast, eDNA metabarcoding provides a more uniform and unbiased species detection range, independent of visibility, behavior, or observer skill (Thomsen and Willerslev 2015, Valentini et al. 2016, Taberlet et al. 2012, Miya et al. 2015).

In recent years, environmental DNA (eDNA) metabarcoding has emerged as a promising tool for advanced marine biodiversity monitoring (Miya 2022; Kumar et al. 2020; Zinger et al. 2019). By detecting trace genetic material from organisms present in environmental samples such as seawater, eDNA metabarcoding provides a non‐invasive, highly sensitive, and observer‐independent method for species detection (Boussarie et al. 2018; Stoeckle et al. 2021; Yamamoto et al. 2017; Ratcliffe et al. 2021). When combined with traditional visual counts, eDNA metabarcoding provides a more comprehensive and robust assessment of biodiversity (Deng et al. 2024), helping to overcome many of the limitations and biases inherent to visual observation. Despite rapid evolution, eDNA metabarcoding approaches still require rigorous in situ validation against independent benchmarks and an explicit assessment of technical and biotic sources of variance. This potential dedicated resource and methodology for effectively monitoring marine biodiversity using eDNA in remote and resource‐limited settings remains scarce. As many experimental designs have been proposed to improve representativeness (Shirazi et al. 2021; West et al. 2020), because each ecosystem has its own specificities (in species richness, temperature), methodological choices must be validated through site‐specific testing before interpreting species detections.

Studies in various reef systems show that eDNA and traditional visual surveys detect overlapping but distinct subsets of fish communities (Boussarie et al. 2018, Mathon et al. 2022, Stat et al. 2017, Pei et al. 2025, Hayes et al. 2025). eDNA tends to reveal cryptic, pelagic, or low‐density species, while visual methods capture more conspicuous and behaviorally observable taxa. Used together, they provide a more complete and complementary view of biodiversity across habitats.

In French Polynesia, reef fish have been extensively surveyed through visual methods, particularly in Moorea and surrounding islands (Planes et al. 2009). However, no peer‐reviewed study to date has combined eDNA metabarcoding with traditional surveys in this region, leaving a gap in integrated biodiversity assessments.

Recent advancements in eDNA applications have driven the development of autonomous filtration systems to support standardized and reproducible sampling (Thomas et al. 2018; Yamahara et al. 2019; Formel et al. 2021). While several filtration systems have been proposed, no consensus exists for large volume sampling under field conditions. Critical sampling parameters have been previously identified such as filtered volume of water (Sepulveda et al. 2019), filter porosity (Barnes et al. 2021; Li et al. 2018), and the number of sampling replicates (Kawakami et al. 2023). In this study, we focused on French Polynesia, a global marine biodiversity hotspot characterized by high endemism, species rarity, and a wide range of reef habitats (Galzin et al. 2023). We used this ecosystem as a model system to develop and validate a comprehensive eDNA‐based monitoring protocol, from in situ water sampling to sequencing and taxonomic assignment, tailored to the specific constraints of remote tropical ecosystems. A particular attention must be dedicated to eDNA collection and preservation as we study tropical reef ecosystems in remote regions, where ambient temperatures are elevated and cold chains are often hard to maintain. To improve the capabilities and reduce limitations of eDNA metabarcoding techniques for assessing marine reef fish biodiversity, we conducted a series of controlled experiments targeting these several critical variables. We also benchmarked a custom autonomous underwater sampler promising for sampling eDNA in tropical regions.

Standard Illumina‐type sequencing platforms are difficult to install and operate in remote regions. Oxford Nanopore Technology (ONT) offers a practical alternative for field‐based sequencing in remote settings, enabling local sequencing with minimal infrastructure using the MinION MK1c (and new version) device. Recent advances in sequencing technology have demonstrated the potential of Nanopore Technology for eDNA‐based studies (Stoeck et al. 2024; Egeter et al. 2022). Its effectiveness for short‐read DNA metabarcoding has been demonstrated (van der Reis et al. 2023; Maggini et al. 2024; Kasmi et al. 2024), and performance relies on robust bioinformatics (Carradec et al. 2020). We implemented and extended the Decona pipeline to improve taxonomic resolution and assignment accuracy in marine eDNA applications (Doorenspleet et al. 2025; Dubois et al. 2024; Baloğlu et al. 2021).

The success of metabarcoding depends on both the choice of the primer used for sequencing and the quality and completeness of reference databases. Incomplete or taxonomically biased reference databases can compromise the accuracy of species identification, particularly for rare or endemic taxa. To ensure consistency and comparability across studies, reference databases must be comprehensive and curated. A regionally relevant and taxonomically resolved database is thus essential. We prioritized building a dedicated, curated 12S mitochondrial reference library for the fish species of the Society Archipelago, based on the MiFish primer, which has proven effective for discriminating fish species in degraded DNA contexts (Miya et al. 2015). Although numerous 12S sequences exist for fish (Iwasaki et al. 2013; Kasmi et al. 2023), gaps and inconsistent annotations remain for Pacific fish, and we worked on a curated, high‐resolution 12S reference database tailored to the fish biodiversity of French Polynesia.

This article explores the importance of developing and implementing an eDNA‐based methodology for the study and monitoring of marine fish biodiversity, with a focus on identifying key sources of variance and testing innovative technologies suited to field deployment. By integrating eDNA metabarcoding technology we aim to establish a monitoring approach that is more objective, observer‐independent, non‐invasive, and holistic, providing essential data for conservation efforts and effective management strategies in the face of environmental changes.

## 2 Materials and Methods

### 2.1 Overview of the Workflow

We designed an end‐to‐end workflow for environmental DNA (eDNA) metabarcoding analysis. It begins with the definition of field‐sampling parameters and the development of an optimized strategy for seawater filtration and eDNA capture in a marine environment. This is followed by a detailed protocol for DNA extraction, PCR amplification, and sequencing of the 12S rRNA gene metabarcoding approach. Finally, the bioinformatics tools and pipeline used to process and analyze the sequencing data are presented.

### 2.2 Sampling Location and Time

Fieldwork was conducted on the coral reef complex around Moorea, Society Islands, French Polynesia, in the South Pacific Ocean (17°32′ S, 149°50′ W). Sampling locations for each experiment are provided in Figure S1. The reef‐lagoon complex supports a diverse range of marine habitats, including fringing reefs, patch reefs, reef flats, and channels, which are home to a high diversity of coral and fish species (Siu et al. 2017).

At three reef habitats along the Tiahura radial in Moorea Island, we collected eDNA samples in triplicate during two seasonal periods (wet season in March and dry season in September). This design enabled a direct comparison of spatial and temporal patterns in biodiversity detection with visual census data collected in the same area and at the same time.

To assess diel variability in eDNA detection, water is filtered in triplicate at 06:00, 12:00, 18:00, and 00:00. To limit auto‐correlation across consecutive days, each time slot is conducted over a month‐long period, with single‐day replicates per time slot.

For the experiment in controlled conditions, seawater samples were collected at the *Te Fare Natura Aquarium*, which reproduces lagoonal conditions representative of the reef crest environment around Moorea Island. This controlled setup was used to validate equipment performance prior to field deployment. Identical water masses were collected with the robot and into 20‐l Nalgene bottles, transported to the next‐door laboratory and filtered using a conventional 142 mm stainless‐steel “tripod” filter holder system (Millipore) in parallel. Triplicate 20 L seawater samples are filtered on 1.2 μm membranes using both systems. The detailed descriptions of this equipment are mentioned below.

This study includes a total of 95 unique eDNA sample replicates collected between November 2021 and March 2023.

### 2.3 Underwater Visual Census (UVC)

Fish communities were assessed by underwater visual censuses along three standardized belt transects (25 × 2 m; 50 m^2^ per replicate) at each site (Galzin and Parravicini 2016). Transects were laid parallel to the shoreline or the reef crest, depending on the habitat (lagoon or outer slope). All UVC surveys were conducted by same‐trained SCUBA divers to minimize observer bias.

### 2.4 Filtration With Robot

**FIGURE 1:**
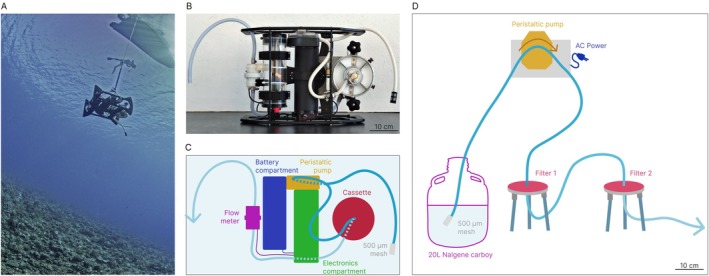
(A) Underwater deployment of the filtration robot, suspended from a boat. (B) Top view of the eDNA filtration module. (C) Annotated components of the device. Seawater is drawn by the pump through the mesh and flexible tubing into the cassette containing up to two filters. (D) Annotated component of the tripod filtration system; two Tripod filtration units are connected in series, allowing sequential filtration using two different pore sizes.

We developed a semi‐autonomous submersible eDNA filtration device (Figure 1) designed to collect and filter seawater in situ, positioning the pump close to the potential eDNA source regardless of depth. This configuration improves DNA capture efficiency and minimizes sample handling losses. The system is powered by a 14.4 V lithium‐ion battery driving a peristaltic pump (nominal flow rate: 1.0 L·min^−1^), with water passing through a removable cassette accommodating one or two 142 mm filters. A 500 μm pore mesh strainer is placed at the inlet, upstream of the pump, to remove large debris. Filtration volumes are precisely controlled via an integrated flowmeter linked to a programmable Arduino controller. The system is operated through a custom Android application, allowing users to preset the target filtration volume, after which the pump automatically stops. All removable filtration components are pre‐assembled in a controlled, DNA‐free environment to reduce contamination risk. The cassette design is compatible with standard Millipore holders, and customizable programming options enable adaptation to diverse field conditions.

### 2.5 Filtration With Tripod

Our filtration system on tripod (Merck‐Millipore) is configured in a sequential setup to collect multiple size fractions (Figure 1D). The setup includes: (1) A pore mesh strainer of 500 μm placed at the inlet, upstream of the pump, to remove large debris; (2) A first 142 mm filter housing one of three interchangeable porosities (0.8, 1.2, or 3 μm) to explore the effect of pore size on eDNA capture; (3) A second 142 mm polycarbonate membrane of 0.2 μm porosity, displayed to retain smaller particles, including microbial and free‐floating DNA. This setup yielded two simultaneous fractions; swapping the middle membrane produced six fractions in total, each filtered in triplicate.

To evaluate how the filtration volume affects species detection efficiency, we filtered 10, 20, and 35 L seawater volumes through 1.2 μm polycarbonate membranes, with each volume tested in triplicate to assess consistency and reproducibility. Filtration capacity is constrained by particular loads, pump flow rate capacity, and the maximum pressure tolerance of the filtration system. In moderately turbid lagoon water, volumes > 35 L proved unfeasible with 1.2 μm membranes, due to increased clogging and pressure buildup.

### 2.6 Contamination Risk Management

To minimize the risk of contamination inherent to eDNA studies, a strict decontamination protocol was applied throughout sample handling and filtration. Prior to each field filtration, by wearing gloves, all reusable equipment was cleaned using a 10% bleach (sodium hypochlorite) solution, followed by a sterile Milli‐Q water rinse to ensure chemical removal and sterility before putting in a sterile bag. Whenever possible, the equipment was handled under a UV‐sterilized laminar flow hood to minimize contamination. Tubes, plastic bags, pipettes, and a single‐aliquot conservation buffer are single‐used for each field sampling. For the filtration system, the mesh strainer and tubing were decontaminated by circulating a 10% bleach solution through the system using the pump until the solution had fully passed through all components. The bleach solution was left in contact with the system for 5 min, after which the system was thoroughly rinsed with a minimum of 5 L of Milli‐Q water to remove residual bleach. These procedures were applied consistently to reduce cross‐sample contamination between different sampling conditions but were not applied between different sampling replicates.

To control our decontamination procedure, a field blank was processed for every field experiment by filtering 1.5 L of commercial water.

### 2.7 Conservation Procedure

Immediately after filtration, each filter is preserved in 5 mL cryogenic storage of DNA/RNA Shield (Zymo Research), a stabilized buffer that prevents DNA degradation. This buffer solution can preserve DNA for several days at 37°C, making it suitable for tropical fieldwork in remote areas where cold‐chain logistics are often unavailable.

### 2.8 DNA Extraction and Purification

Environmental DNA filters were cut into small fragments using a sterile scalpel and transferred into 50 mL Falcon tubes containing the DNA/RNA Shield storage solution and Zymo Bashing Beads (0.1 and 0.5 mm). The samples are vortexed for 1 min, then centrifuged for 1 min at 3000 g.

DNA was extracted using the Quick‐DNA Fecal/Soil Microbe Miniprep Kit (Zymo Research), following the manufacturer's protocol, optimized for marine filter samples as follows. 1200 μL of Genomic Lysis Buffer and 600 μL of 95% ethanol are added to the samples. They are vortexed for 5 s and centrifuged for 1 min at 3000 *g*. The supernatants are collected and transferred to a 5 mL tube. The lysate is loaded onto a Zymo‐Spin IICR column in 800 μL increments, centrifuging for 1 min at 10,000 *g* after each load, and discarding the flow‐through. Then, 200 μL of DNA Pre‐Wash Buffer is added to the column, incubated for 5 min at room temperature, and centrifuged for 1 min at 10,000 *g*. Next, g‐DNA Wash Buffer is added, incubated for 1 min, and centrifuged for 1 min at 10,000 *g*. The Zymo‐Spin IICR column is then placed into a clean 1.5 mL microcentrifuge tube, and 100 μL of DNA Elution Buffer is added for elution. Eluted DNA is subsequently purified using a Zymo‐Spin IIIHCR column placed into a collection tube. A total of 600 μL of Prep Solution is added to the column, followed by centrifugation for 3 min at 8000 *g*. The Zymo‐Spin IIIHCR column is then transferred to a 1.5 mL DNA LoBind tube. The eluate from a 30 s centrifugation of the Zymo‐Spin IICR column at 10,000 *g* is transferred onto the Zymo‐Spin IIIHCR column. A final centrifugation is performed at 16,000 *g* for 3 min. DNA concentration was quantified using Qubit fluorometric quantification, High Sensitivity kit (Thermo Fisher Scientific). Here, field blank control serves as extraction blank control, every blank has shown “Too low” measure with the Qubit High Sensitivity kit.

### 2.9 PCR Amplification and Sequencing

In order to validate primer performance and pipeline accuracy, we built a mock community of reef fish species belonging to three distinct families to avoid any taxonomic ambiguity: *Chaetodon trichrous* (Chaetodontidae), *Centropyge bispinosa* (Pomacanthidae), and *Zebrasoma scopas* (Acanthuridae). DNA extractions on muscle or skin tissues are performed using the Maxwell 16 MDx Instrument (Promega). An additional single‐species positive control containing only *Ellochelon vaigiensis* is also processed similarly. UltraPure water serves as the negative (blank) PCR control.

The PCR is conducted using the MiFish‐U‐F 5′‐GCCGGTAAAACTCGTGCCAGC‐3′ and MiFish‐U‐R 5′‐CATAGTGGGGTATCTAATCCCAGTTTG‐3′ primer set (Miya et al. 2015) which is designed to target a hypervariable region (*ca*. 170 bp) of the mitochondrial 12S rRNA. To index individual filter replicates (biological samples), up to 24 unique ONT barcodes are used, both ligated to forward and reverse primers, thus enabling pooling (i.e., multiplexing) and optimization of sequencing runs. PCR reactions (50 μL) included 25 μL 2× Qiagen Multiplex Master Mix, 5 μL primer mix (F/R, equimolar in TE 10:1, up to 100 μM), 10 ng template DNA. The thermocycler conditions were set up as follows: initial denaturation at 95°C for 15 min, 35 cycles of (1) denaturation at 94°C for 30 s, (2) annealing at 54.2°C for 90 s, and (3) extension at 72°C for 90 s, finally followed by final extension at 72°C for 10 min. For samples requiring reduced non‐specific amplification (e.g., 16S off‐targets; (Kawato et al. 2021)), the annealing temperature was set to 60.2°C. In order to evaluate the impact of PCR replicates on biodiversity detection, a set of independent PCRs is conducted on the same DNA extraction. The number of PCRs performed is either one, three, five or ten, and the resultant products are then pooled prior to sequencing. In all other experiments, three PCR replicates were carried out.

PCR products are purified with AMPureXP beads (Beckman Coulter), quantified with the Qubit HS kit (Thermo Fisher Scientific), and visualized using 2% E‐Gel EX agarose gels on the E‐Gel Power Snap system prior sequencing.

Sequencing is performed on Oxford Nanopore Technologies (ONT) R10.4.1 flow cells, using the SQK‐LSK114 ligation sequencing kit, following the manufacturer's protocol. Runs are conducted for 48 h at 450 bases per second, yielding sufficient read depth to cover the diversity present in the samples.

### 2.10 Nanopore Read Processing

#### 2.10.1 Basecalling and Demultiplexing

The raw .*fast5* files are basecalled using the Super Accurate *Guppy* (SUP) algorithm. *Porechop* v0.2.4 (https://github.com/rrwick/Porechop) is used for demultiplexing, offering more effective trimming than *Guppy*. Only reads with identical barcode pairs on both ends are retained to minimize chimeric artifacts (Alberdi et al. 2018). The reads are then filtered to retain only those with a Qscore > 10, a minimum length of 180 bp, and a maximum of 250 bp, in order to exclude non‐specific 16S amplification products.

#### 2.10.2 Operational Taxonomic Unit (OTU) Assessment

Given the limited availability of tools optimized for Nanopore‐derived eDNA reads, we chose *Decona* over *Proname* because it offers the possibility to perform an additional clustering round in the pipeline, which is necessary for our datasets (Doorenspleet et al. 2025). *Decona* starts with clustering reads with the *cd‐*hit algorithm (Fu et al. 2012) with 97% sequence identity to maintain species‐level resolution (Alberdi et al. 2018; Miya et al. 2020). Then, *Decona* calls *minimap2* to align sequences to a reference and uses *medaka* and *racon* to polish and get a consensus sequence for each reference. The *Decona* framework was expanded through the integration of custom Python and Bash scripts facilitating a secondary 97% clustering process with *cd‐hit* on polished consensuses from all runs of an experiment. This approach resulted in the generation of an OTU result file, encompassing a consensus sequence and the corresponding taxonomic classification with BLAST metrics, presented in tabular format for in‐depth analysis of OTU distribution through samples.

#### 2.10.3 Taxonomic Assignment of 12S OTUs

A custom 12S rRNA gene reference database for French Polynesian fishes is constructed to improve the taxonomic assignment. A total of 190 12S sequences were generated via Sanger sequencing from voucher specimens previously collected around Moorea Island. We completed this resource with 216 12S rRNA sequences retrieved from NCBI GenBank, prioritizing Sanger sequences when available. All sequences are manually curated to remove primer regions and to ensure consistent taxonomic annotation. The final database includes 431 fish (*Actinopterygii*) sequences covering 62 families, and despite representing approximately 59% of the fish species listed in shore Fish of French Polynesia (Siu et al. 2017) in Moorea Island, these species correspond to 97% of species recorded at least two times through visual surveys around the island of Moorea since 2004 as recorded in the Service National d'Observation Corail (SNO Corail) annually. The dataset also includes 15 *Chondrichthyes* species and a human mitochondrial fragment sequence matching with MiFish primers for contamination control. The complete database is available in the project's public repository (https://doi.org/10.5281/zenodo.16969829).

### 2.11 OTUs Table Analyses

#### 2.11.1 OTUs Curation Process

OTU table analyses are performed on R 4.2.2 version. First, reads affiliated to contaminants (Human, bacteria) or the positive control ( *Moolgarda engeli* ) were removed. Second, to prevent false positive, we use the “decontam” R package (v 1.30.0) (Davis et al. 2018). *isContaminant()* function with 0.5 threshold, identify contaminants by the prevalence of OTU in blank control (filed/extraction and PCR) in regard of their prevalence in true environmental samples. Then, to account for uneven sequencing depth across samples, the OTU table is rarefied using the rarefy function from the vegan package and rarecurve, normalizing all total number of reads per sample to 90% of the smaller reads number sample. For direct comparison with visual census data, OTUs are aggregated at the species level. Finaly, for each species, BLAST bit scores are averaged across all associated OTUs, with weighting based on the number of reads per species. Taxa with a mean bit score below 250 are classified as “unknown”.

#### 2.11.2 Statistical Analyses

*Barplots* are generated with ggplot library and the accumulation curve is generated with vegan, specaccum function. Species composition and richness evaluation are performed with phyloseq library. Jaccard distance matrix is computed on presence/absence data with vegdist function of vegan package. Mann–Whitney‐Wilcoxon test is used to evaluate differences in richness metrics between groups. To visualize community differences on an ordination scale, PCOA are performed with cmscale function, and the homogeneity of beta dispersion with betadisper function from vegan package. PERMANOVA is used to assess which of Habitat or sampling season explained the most variance in beta diversity with the following command line: vegan::adonis2(dist ~ Habitat * Month, data = sample_dist, permutations = 10,000).

## 3 Results

### 3.1 Impact of the Membrane Porosity on Fish Detection

**FIGURE 2:**
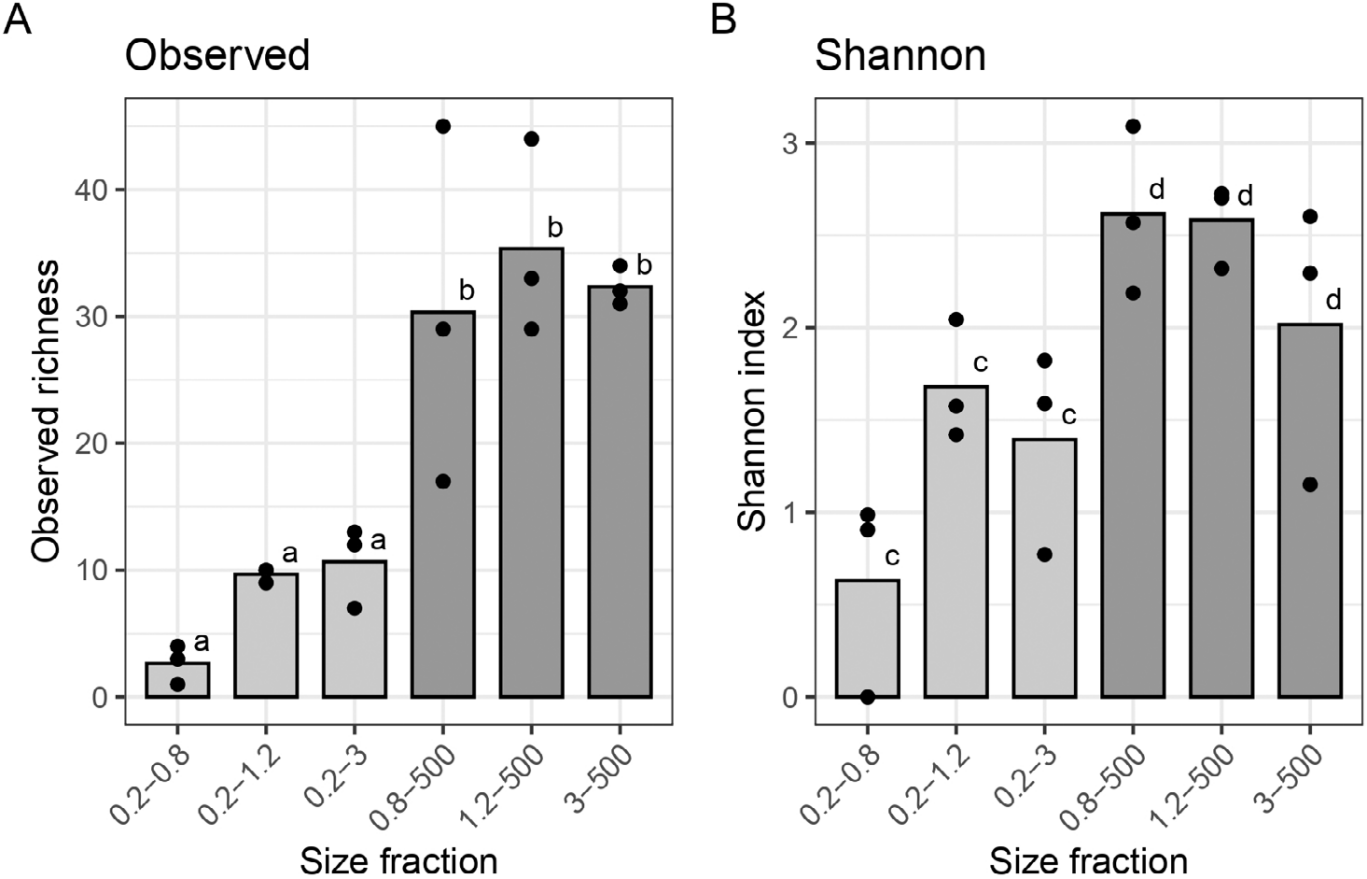
Alpha diversity metrics for each size fraction. Bars represent group means and dots represent individual replicates (*n* = 3 per group) (A) Richness (Number of OTUs). Mann‐Withney‐Wilcoxon test shows a significant difference between a and b groups with a *p*‐value of 0.00041. (B) Shannon index. Mann‐Withney‐Wilcoxon test shows a difference between the two groups (c and d) with a *p*‐value of 0.00078.

Environmental DNA samples coming from Moorea/Papetoai lagoon environment were filtered through different pore sizes. Alpha diversity varied markedly across size fractions, with observed richness ranging from 1 to 47 OTUs and Shannon diversity index values from 0 to 3.1, indicating substantial heterogeneity in fish community composition (Figure 2A,B). Size fraction had a significant effect on diversity, with the 1.2 μm filter size capturing the highest mean species richness. This suggests that this size range concentrates a greater diversity of fish species. Prior studies showed that 0.2 and 0.45 μm filters are particularly effective for capturing bacterial and extracellular DNA. Among the tested porosities, 1.2 μm membranes offered the best size fraction for detecting fish eDNA with limited bacteria DNA capture.

### 3.2 Efficiency of the Automated Filtration System

**FIGURE 3:**
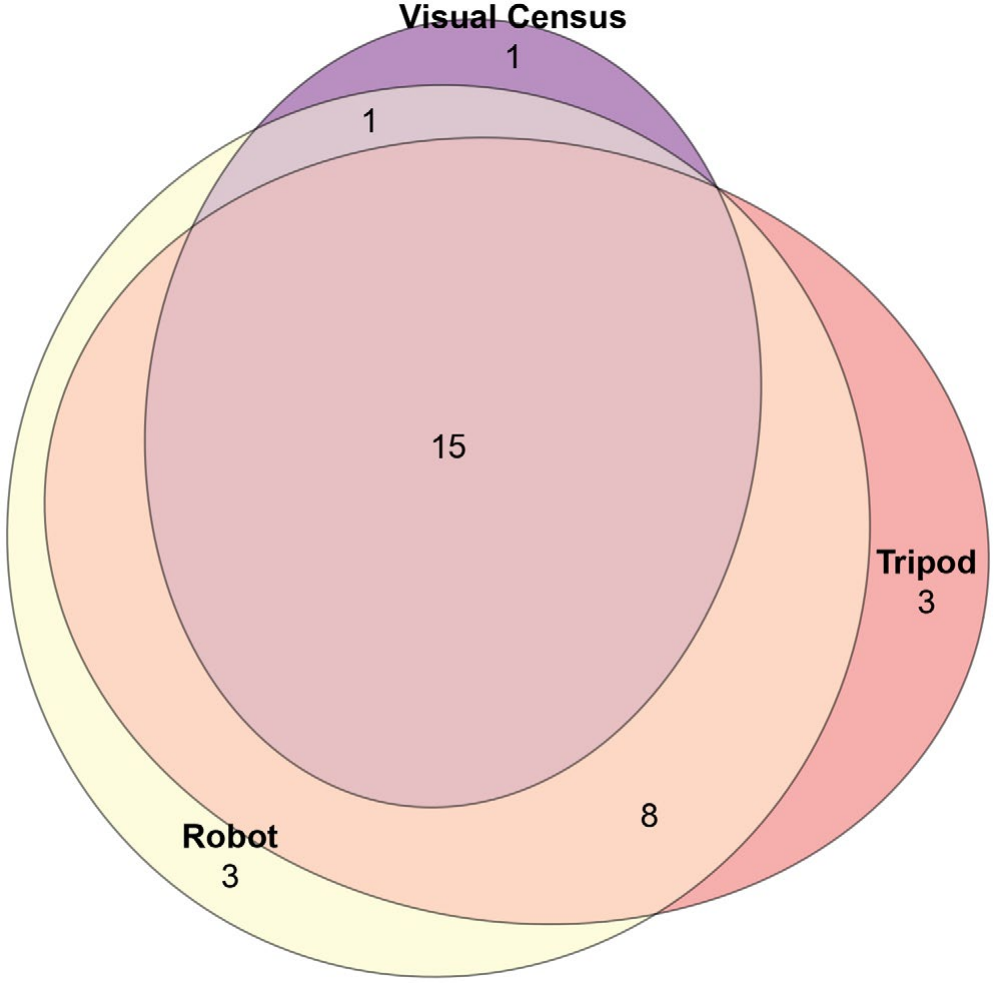
Number of shared species between different sampling methods.

The comparison between sampling methods in an aquarium environment reveals both overlaps and discrepancies in species detection. All observed species were also detected by the robot‐based eDNA metabarcoding approach (Table 1). Because *Pomacentrus pavo* and *P. coelestis* have the same 12S sequence, they are indistinguishable with the eDNA approach. The corresponding OTU is thus labeled “ *Pomacentrus pavo* ‐coelestis,” which is consistent with the presence of *P. pavo* in the DNA samples. Identically, 12S sequences of *Acanthurus pyroferus* , *Acanthurus reversus* , and *Acanthurus olivaceus* are very similar and introduce bias in the detection of these species by eDNA. In total, 14 taxa not recorded in visual surveys were identified with the eDNA approach (Table 1 and Figure 3).

**TABLE 1:** Taxa table of (1) cumulative relative abundance of number of reads DNA from environmental sample and (2) relative abundance of number of species observed by visual census in the aquarium.

Taxon	Robot			Tripod			Visual census	Family	Order	Class
	1	2	3	1	2	3				
*Valenciennea strigata*	17.462	17.05	13.834	15.52	19.464	21.502	7.895	Gobiidae	Gobiiformes	Actinopteri
*Asterropteryx semipunctata*	0.076	0.126	0.05		0.306					
*Parupeneus multifasciatus*			0.018					Mullidae	Syngnathiformes	
*Myripristis adusta*		0.078						Holocentridae	Holocentriformes	
*Neoniphon sammara*			0.032							
*Pterocaesio tile*				0.116				Lutjanidae	Lutjaniformes	
*Ctenochaetus flavicauda*	6.142	5.528	6.368	6.224	8.192	6.622	2.632	Acanthuridae	Acanthuriformes	
*Zebrasoma scopas*	2.918	2.446	2.508	4.952	2.484	1.128	2.632			
*Acanthurus pyroferus*		0.004					2.632			
*Acanthurus reversus* −*olivaceus*	1.576	2.522	1.624	2.344	0.878	1.218				
*Chaetodon citrinellus*	23.968	16.926	16.36	25.714	33.614	24.972	2.632	Chaetodontidae	Chaetodontiformes	
*Heniochus chrysostomus*	2.318	2.754	3.734	2.256	2.256	0.728	2.632			
*Chaetodon trichrous*	0.084	0.148		3.61	1.506	0.596				
*Bodianus axillaris*	6.194	16.268	21.064	4.148	2.998	2.656	2.632	Labridae	Labriformes	
*Halichoeres trimaculatus*	7.77	10.22	7.234	8.178	7.216	5.256	7.895			
*Pseudocheilinus hexataenia*	0.04	0.16	0.056	0.284	0.068	0.02	7.895			
*Labroides dimidiatus*	0.54	0.518	1.94	1.332	0.456	0.664	5.263			
*Cirrhilabrus scottorum*	0.18	0.122	0.04	0.178	0.27	0.216	2.632			
*Chlorurus sordidus* −*spilurus*	0.112	0.034		0.13		1.546				
*Centropyge flavissima*	0.348	0.744	0.51	0.262	0.746	1.02	5.263	Pomacanthidae	Perciformes	
*Centropyge bispinosa*	0.226	0.098		1.8	0.202	0.118				
*Chromis viridis*	14.534	9.046	8.726	13.262	11.96	21.472	13.158	Pomacentridae		
*Dascyllus aruanus*	4.878	6.136	6.906	1.8	2.748	6.826	15.789			
*Pomacentrus pavo*							10.526			
*Pomacentrus pavo* −*coelestis*	6.03	4.426	6.348	5.57	1.376	1.98				
*Amphiprion chrysopterus*	0.968	1.316	0.91	0.3	0.564	0.32	5.263			
*Dascyllus trimaculatus*	2.138	1.3	0.566	0.364	1.43	0.12	2.632			
*Chrysiptera brownriggii*	1.356	1.492	1.036	0.544	0.57	0.458				
*Chromis acares*				0.154						
*Carcharhinus melanopterus*				0.896	0.528	0.098		Carcharhinidae	Carcharhiniformes	Chondrichthyes
unknown	0.142	0.538	0.136	0.062	0.168	0.464		Unknown	Unknown	Unknown

**FIGURE 4:**
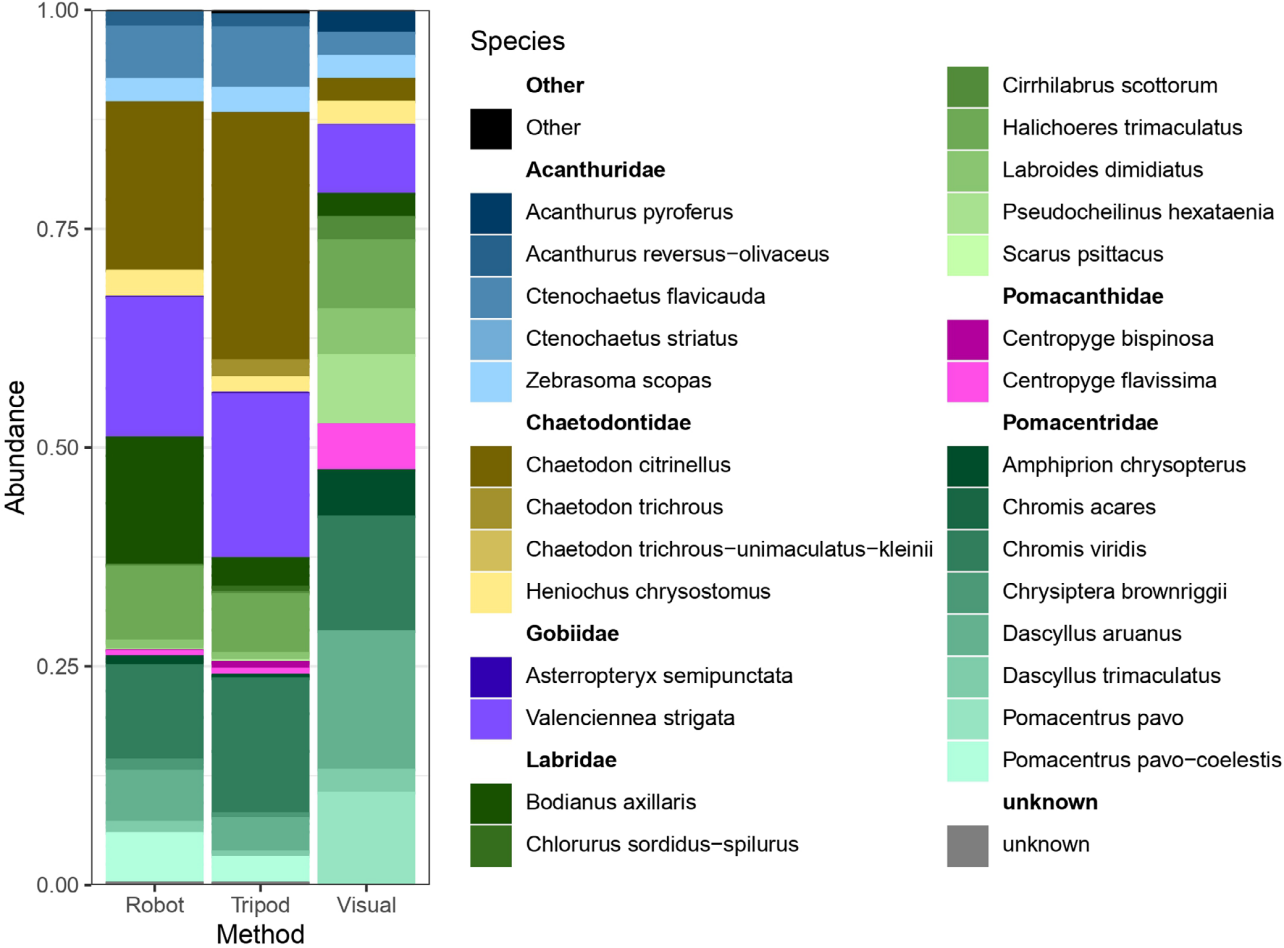
Distribution of relative read counts (eDNA) and relative visual counts by fish species. The three sampling replicates are cumulated here. Only the 21 most abundant species are represented here. The 9 other species are grouped into “Other” section and are detailed in Table 1.

Relative read counts from eDNA metabarcoding and visual abundance estimates show broadly consistent biodiversity patterns across sampling methods. The autonomous underwater filtration system and the tripod‐based method yield similar results, with no significant differences in species richness indices (Observed, Shannon, Chao1, and Simpson; Wilcoxon tests *p* > 0.2), indicating comparable efficiency in biodiversity recovery. In contrast, clear differences emerge between eDNA‐based methods and visual census: Shannon alpha diversity is significantly higher in eDNA samples (ANOVA, *F* = 23.59, *p* < 0.05), and relative abundance distributions also differ significantly between approaches (Wilcoxon test, *W* = 3029.5, *p* < 0.005), suggesting that eDNA metabarcoding methods may detect more diverse and/or differently represented communities than visual surveys (Figure 4). The species detected by eDNA but not observed visually represent a very small proportion of reads (0.5% on average) compared to species common to both methods (5.7% on average). This demonstrates the potential of eDNA to provide quantitative representation of biodiversity.

### 3.3 Optimal Filtration Volume for Increasing Accuracy in Biodiversity Estimates

**FIGURE 5:**
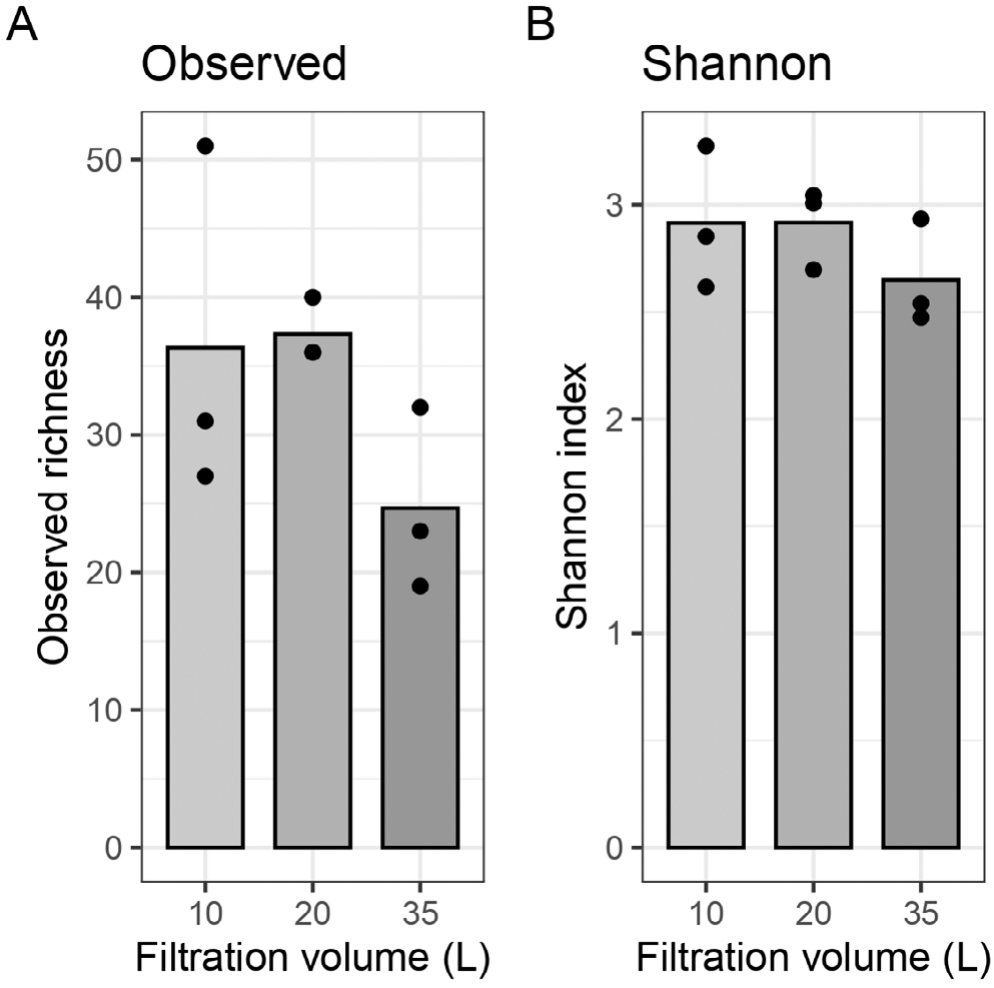
Alpha diversity measured across filtration volumes. (A) Observed Species. (B) Shannon index.

This experiment helped identify a practical trade‐off between sampling effort and biodiversity detection, guiding standardized protocols for marine eDNA surveys under field conditions, at the Moorea/Papetoai sampling location, in the lagoon environment. To understand the impact of water volume, the alpha diversity was measured after 10, 20, and 35 L of filtered water through 1.2 μm membranes. No significant differences (Mann‐Withney‐Wilcoxon test, *p* > 0.05 for all paired comparisons) were observed between the 10 and 20 L for either species richness or Shannon index, indicating that doubling the volume within this range does not impact diversity estimates. However, we observed a greater variability in richness between our 10 L replicates (SD = 12.86) than between our 20 L replicates (SD = 1.53). Surprisingly, both metrics declined with 35 L, suggesting a potential decrease in detectable diversity at higher filtration volumes. This pattern may reflect filter clogging, DNA degradation, or the accumulation of inhibitory compounds, ultimately reducing eDNA metabarcoding recovery efficiency. Together, these results indicate that filtering beyond 20 L can decrease biodiversity recovery under the conditions tested (Figure 5).

### 3.4 Appropriate Sampling Effort for Representative Diversity Estimates

**FIGURE 6:**
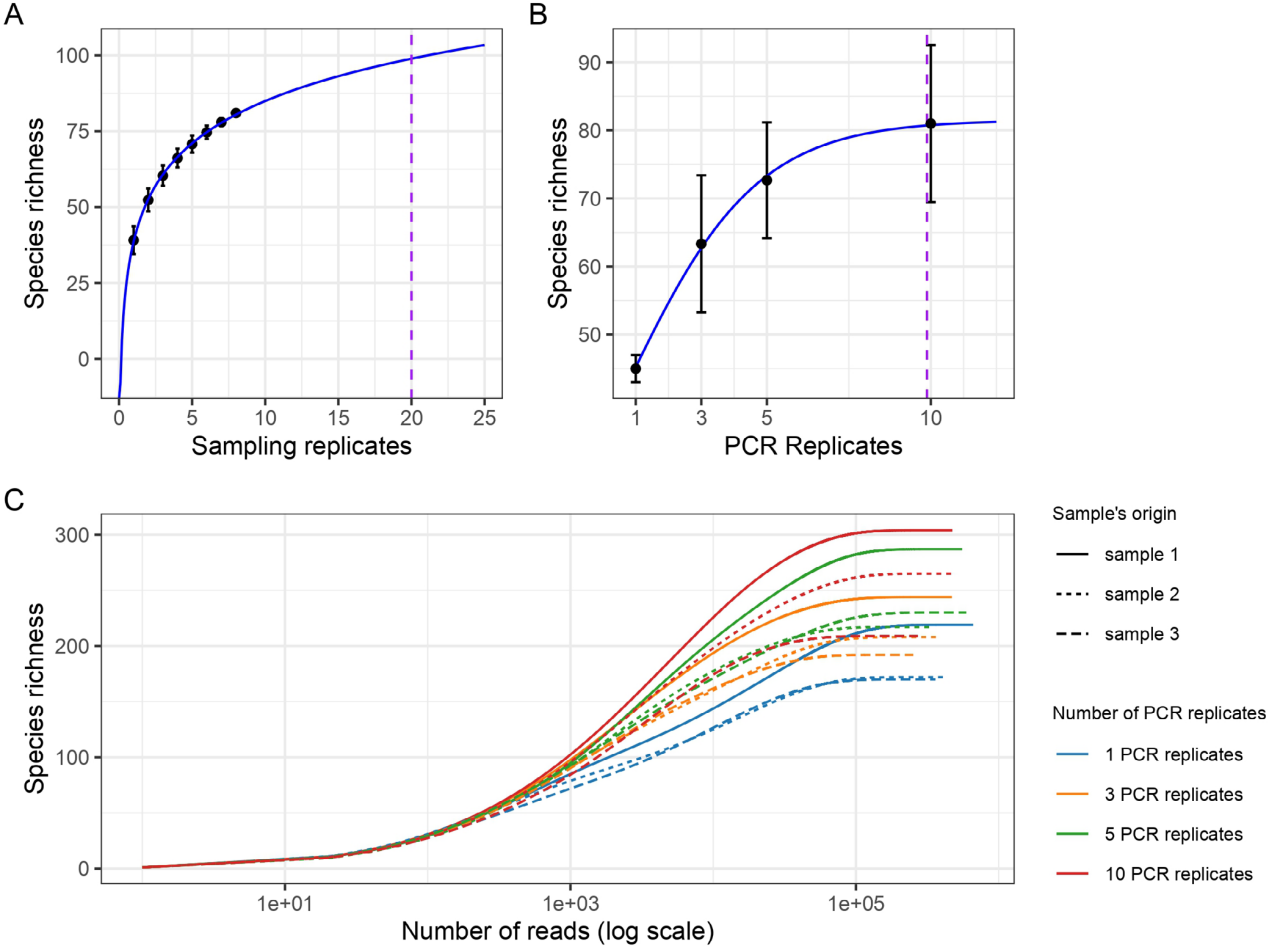
(A) Species accumulation curve based on one to eight filtration replicates. For each number of sampling replicates, the mean cumulative number of species across the eight samples is represented by a dot, with standard deviation shown as error bars. The blue line represents a logarithmic regression, and the dashed line indicates the number of replicates beyond which the gain in detected species is less than one. (B) Effect of PCR replicates on species richness. Mean value for 3 sampling replicates in each PCR pool are associated with standard error bar. The dashed line represents the number of PCR replicates at which 99% of the plateau is reached, corresponding to approximately 10 cumulative PCR replicates. (C) Rarefaction curves showing sequencing depth for samples of varying richness, with the *x*‐axis on a logarithmic scale. Linear scale axis is provided in Figure S3.

To determine whether three sampling replicates sufficiently capture local reef fish biodiversity, we filtered up to eight replicate filtrations of 20 L seawater collected at a single site near the shoreline in lagoon environments. By analyzing taxonomic accumulation curves, we assessed the optimal sampling effort required to ensure robust and cost‐effective biodiversity assessments. We show here that the number of observed species increased continuously with additional samples, indicating that a higher sampling effort would continue to add diversity (Figure 6A). However, beyond approximately 20 replicates, adding more samples contributes less than one additional species, suggesting a tendency towards saturation. Based on these results, three replicates recovered ~60% of a theoretical total diversity, which is a solid baseline.

To assess whether additional PCR replicates enhance biodiversity recovery we performed 1, 3, 5, and 10 independent amplifications of three different DNA samples. The number of species detected increased notably with the number of PCR replicates, rising from an average of 45 species with a single replicate to approximately 81 species with 10 replicates (Figure 6B). The trend shows a rapid increase between 1 and 5 replicates, followed by a leveling off between 5 and 10 replicates, indicating increasing PCR replicates effort will tend to a richness detection saturation. In contrast, the Shannon diversity index remained stable across different numbers of PCR replicates, with mean values ranging from 2.04 to 2.08 (standard deviation: ~0.05–0.10; Figure S2). This indicates that community evenness and overall structure are largely unaffected by replicate number. While increasing the number of PCR replicates improves the detection of rare species—as reflected in higher observed richness—it does not substantially influence the overall community diversity as shown by Shannon's index.

To test the required sequencing depth, we performed rarefaction curves for several samples of different richness. All samples reach a plateau with only 10,000 reads, indicating that a low sequencing depth is sufficient to capture the specific diversity present in the samples whatever the richness (Figure 6C). This result confirms a sufficient sequencing depth and indicates that differences across treatments reflect true biological or methodological variation rather than sequencing effort.

### 3.5 Comparing eDNA Metabarcoding and Visual Census Approaches in Coral Reef Environment

**FIGURE 7:**
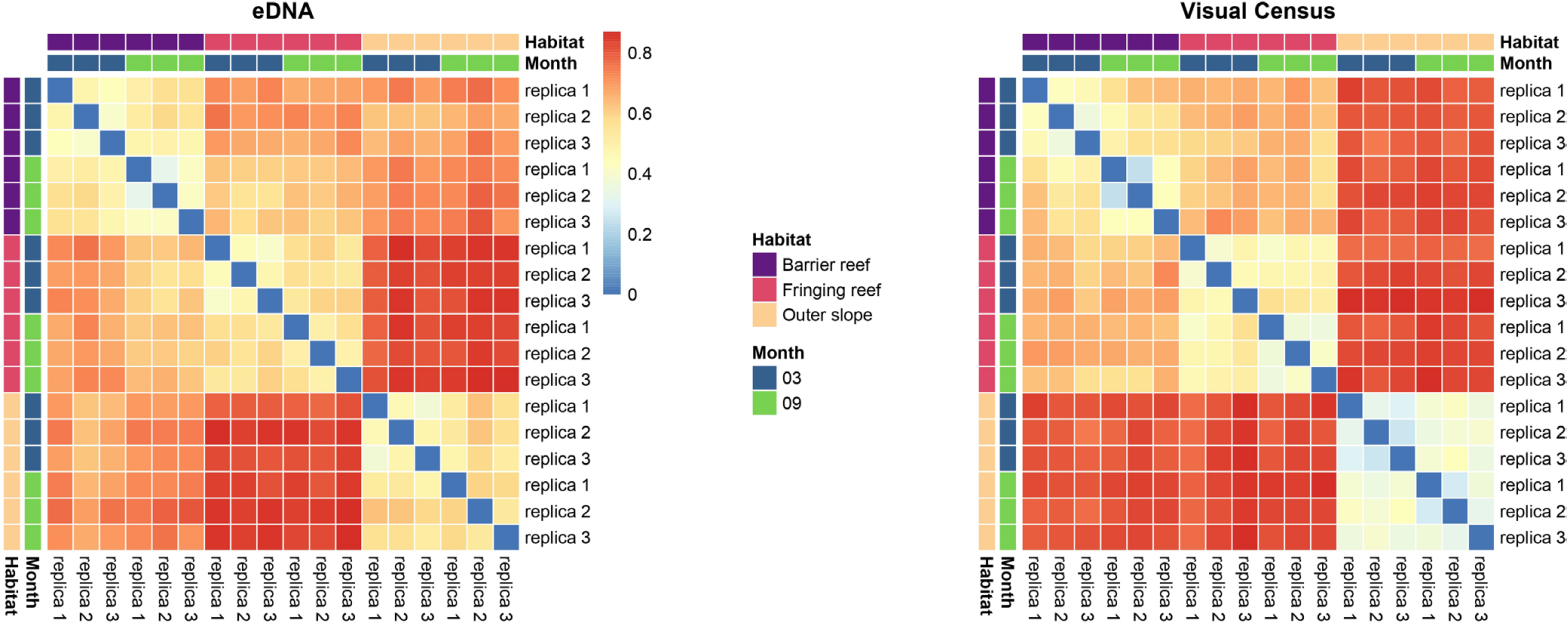
Jaccard distance matrices between different sampling locations and seasons. Relative abundance data were converted to presence/absence matrices to compare community compositions across eDNA samples (left) and visual census (right). Reddish colors indicate greater dissimilarity between samples.

Environmental DNA samples are achieved with the submersible pump in different habitats of the reef on the same radial, combining with visual fish census, in March and September. Both Jaccard distance matrices (visual and eDNA‐based) indicate that the two lagoon communities (barrier and fringing reefs) are more similar to each other (mean distance = 0.65 for both methods, and individually) than to the outer slope community (mean distance = 0.80 overall, 0.82 for UVC, and 0.77 for the eDNA‐based method) (all Wilcoxon tests, *p* < 0.05; Figure 7). These results confirm a consistent pattern across both methods: the outer slope harbors a more distinct fish community relative to lagoonal habitats, while intra‐lagoon communities are comparatively more similar. However, when using environmental DNA (eDNA) metabarcoding, outer slope communities appear less distant compared to visual census data, suggesting some degree of cross‐habitat eDNA transport or broader spatial integration. Specifically, 10% of the species detected in barrier reef eDNA samples are species exclusively associated with the outer slope habitat (18 species out of 180), whereas in the reverse direction, only 1%–2% of species appeared translocated (2 out of 145). Site‐specific species are evaluated by analyzing the occurrence of fish species presence over 20 years based on fishes communities annual monitoring around Moorea Island and through the 3 reef habitats.

**FIGURE 8:**
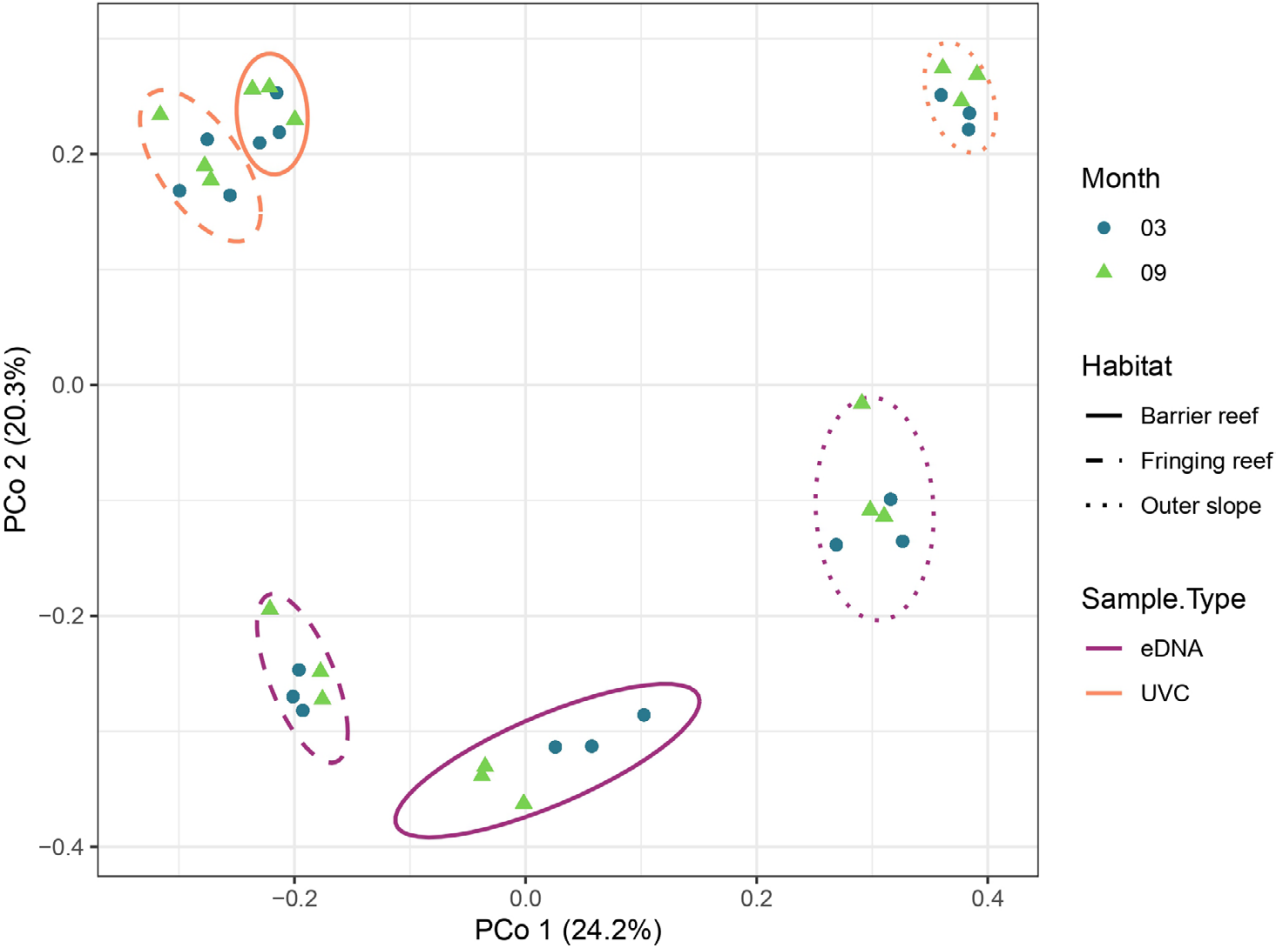
Principal Coordinates Analysis (PCoA) based on Jaccard distance matrices computed from presence/absence data for eDNA and visual census methods. Samples from the same habitat are enclosed by lines with different styles: Solid lines for barrier reef, dashed lines for fringing reef, and dotted lines for outer slope habitats. Line color indicates the sampling method: Purple for eDNA and orange for visual census (UVC). Sampling months are represented by symbols: Green triangles for September (09) and blue circles for March (03).

PERMANOVA analyses of Jaccard distances showed that habitats explained most of the variance of community dissimilarity (*R* ^2^ = 49%, *p* < 0.0001 for eDNA; *R* ^2^ = 62%, *p* < 0.0001 for the visual census), with a significant seasonal effect (*R* ^2^ = 6%, *p* = 0.0345 for eDNA; *R* ^2^ = 5%, *p* = 0.0092 for the visual census) and in interaction between seasons and habitats (*R* ^2^ = 13%, *p* = 0.0092 for eDNA; *R* ^2^ = 11%, *p* = 0.0109 for the visual census). PERMANOVAs validates that both methods (i.e., eDNA metabarcoding and visual census) converged on similar partitioning of the variance in Jaccard distances. Principal Coordinates Analysis (PCoA) based on Jaccard distances revealed clear patterns of habitat structuring in reef fish communities, with distinct clustering according to habitat type (Figure 8). This visual confirmation supports the strong influence of habitat on community composition detected through eDNA metabarcoding.

### 3.6 Assessing 24 h Temporal Variability of Reef Fish Biodiversity With eDNA Metabarcoding

**FIGURE 9:**
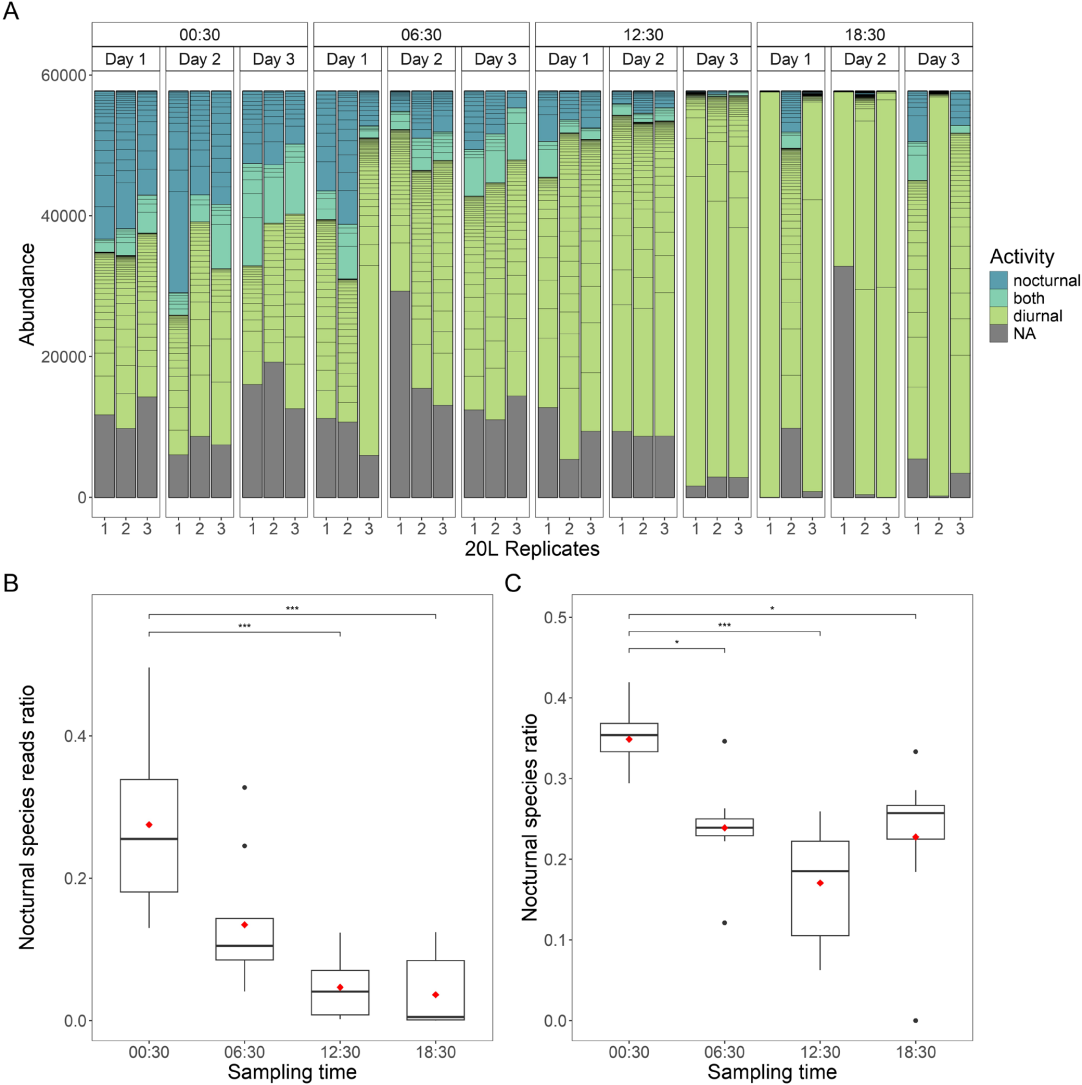
(A) Variations between diurnal or nocturnal species. Each set of three sampling replicates is shown separately. Samples are grouped first by sampling time, then by sampling day. Boxplots displaying (B) read ratios of fishes species actives at night and (C) nocturne species ratio across different sampling times. A red diamond marks the mean value for each time point. Stars indicate significants differences between sampling times (Kruskal–Wallis test, * *p* < 0.05 and *** *p* < 0.001).

The stability of eDNA in natural environments was investigated by analyzing variations in species abundance throughout the diurnal cycle. In the present study, species activity traits were extracted from Parravicini et al. (2021) in order to compare the occurrence of fish species found with eDNA metabarcoding with their activity. A likelihood ratio test comparing a generalized linear mixed model (GLMM) to a simpler generalized linear model (GLM) showed no significant effect of sampling day (LRT = 2.12, *p* = 0.145), thereby justifying the processing of the 3 days as technical replicates alongside the 20 L filtration replicates (Figure 9A).

Subsequent Kruskal–Wallis tests revealed significant differences in nocturnal species read ratios across sampling times (*χ* ^2^ = 22.39, *p* = 5.42 × 10^−5^). Dunn's post hoc tests indicated significant pairwise differences between 00:30 and 12:30 (*p* = 0.00065), and between 00:30 and 18:30 (*p* = 0.00019) (Figure 9B). Similarly, analysis of nocturnal species ratios showed a consistent temporal pattern (*χ* ^2^ = 21.01, *p* = 1.05 × 10^−5^). Post hoc comparisons revealed significant differences between 00:30 and 12:30 (*p* = 4.35 × 10^−5^), 00:30 and 18:30 (*p* = 0.0251), and 00:30 and 06:30 (*p* = 0.0195) (Figure 9C). These findings reveal the temporal structure in fish diversity over the diel cycle, with nocturnal species enriched at night and diurnal species dominating daytime samples. This pattern suggests that fish behavior directly influences the release dynamics of environmental DNA into the water column. These findings highlight the high temporal sensitivity of the eDNA approach, which appears capable of capturing community‐level variations over just a few hours, despite continuous water renewal in the lagoon system.

## 4 Discussion

This study aimed to design, test and validate an end‐to‐end eDNA‐based fish species monitoring framework. Unlike most eDNA applications, which are optimized for temperate or inland systems, our framework is adapted to the constraints of tropical reef environments in remote regions. The present study focuses on defining both technical and biotic sources of variance that influence the reliability and resolution of eDNA‐based biodiversity assessments. These objectives guided the experimental evaluation of key methodological components, ranging from filtration parameters to PCR replicates and sequencing strategy. In addition, the capacity of eDNA metabarcoding to reflect the spatial, temporal, and ecological dimensions of reef fish communities was assessed. The findings of this study quantify the variability arising from technical factors and environmental factors, thereby demonstrating the robustness of the approach relative to traditional survey methods. Among the key results we can highlight that: (1) Filtering 20 L on 1.2 μm membranes maximized detections; (2) the use of a submersible, programmable sampler matched laboratory tripod results while streamlining logistics and limiting contamination; (3) three field replicates captured ≈60% of local richness, whereas extra PCR replicates chiefly retrieved rare OTUs and kept Shannon diversity stable beyond 3–5; and (4) eDNA captured daily variations in biodiversity with nocturnal taxa peaking at night and diurnal taxa by day. In the end we argue that eDNA recovered spatial, seasonal, and diel signals that are comparable to, and often more extensive than, those obtained through visual surveys.

### 4.1 Filtration Protocol: Pore Size and Volume

One of the primary goals was to identify a robust filtration protocol. The option to choose the pore size provided by the 142 mm polycarbonate membrane filters (Merck‐Millipore) was a key factor in adjusting our sampling protocol to local environmental conditions, which led us to prefer this method to capsule filters, like Sterivex or Waterra type. The results of this study highlight the significance of membrane porosity in optimizing fish biodiversity detection using eDNA. The experiments clearly showed that 20 L of seawater filtered through a 1.2 μm membrane offers the optimal compromise between operational feasibility and biodiversity detection. This pore size allows retaining a wide range of eDNA particle sizes. This capture efficiency is governed by a pore size–throughput trade‐off (Barnes et al. 2021; Eichmiller et al. 2016; Brandão‐Dias et al. 2023).

Filtering a large volume is motivated by the high fish richness of the ecosystem. However, the decline in diversity with 35 L samples suggests that large volumes may result in filter saturation, DNA degradation, or retention of inhibitors. This is a significant consideration when developing sampling protocols under field conditions. The decline in richness at 35 L is consistent with multiple mechanisms reported in optimization studies. Progressive clogging, which reduces effective flow through the active surface, can result in extended run times, which can lead to an increase in co‐concentration of humic substances or other PCR inhibitors (Eichmiller et al. 2016). Collectively, these results support moderate volumes (10–20 L) paired with replicate samples rather than a few very large filtrations, as a robust baseline for reef settings (Liu et al. 2024; Sepulveda et al. 2019).

### 4.2 Standardizing Field Sampling with Autonomous Submersible Filtration

The employment of an autonomous underwater filtration system, positioning the pump at depth, close to the DNA source, improves sampling standardization, limits handling steps, and lowers contamination risks, key advantages repeatedly highlighted for autonomous samplers (Yamahara et al. 2019; Formel et al. 2021; Jones et al. 2024). A side‐by‐side comparison with traditional tripod filtration confirmed that robot‐based sampling matches in species detection and is even superior in replicability. This sampling device offers four key benefits for eDNA metabarcoding studies. Firstly, it enables the filtration of large volumes within a single sampling replicate, ensuring consistency across replicates. Secondly, it allows filtration through two filters in series, enabling the analysis of both microbial and eukaryotic communities. Thirdly, it facilitates sampling in remote areas by simplifying field operations, thereby optimizing costs, time, and carbon footprint. Finally, it encourages the involvement of local stakeholders in scientific data collection, requiring only minimal training.

### 4.3 Replication Strategy

The data analysis demonstrates that field replication is the primary driver of increase in richness (approximately 60% with three replicates of 20 L), while PCR replication enhances the detection of rare amplicons without affecting community evenness. In fact, community structure metrics (e.g., Shannon index) remained stable beyond 3–5 replicates. This plateau indicates that diversity saturation occurs quickly in terms of community evenness. However, the benefits of additional amplification for the detection of rare species are evident. This asymmetry is consistent with meta‐analyses and experimental work: stochasticity in both the environment and the PCR process produces false negatives, and replication reduces that risk, but community‐level metrics stabilize after a few PCR repeats (Ficetola et al. 2015; Furlan et al. 2016; Shirazi et al. 2021). However, this raises a trade‐off between cost, effort, and gain in richness. It also confirms that eDNA‐based methods are sensitive to stochastic PCR effects, which complicates quantitative interpretations and challenges the assumption that read counts directly correlates with species abundance. When quantitative interpretation is critical, targeted qPCR/ddPCR calibration for focal species remains advisable (Furlan et al. 2016; Shelton et al. 2022). In the end, and in line with our results and earlier works, we recommend ≥ 3 field replicates per site and 3 technical PCR replicates per sample as a default; increasing to five PCR replicates is warranted when maximizing rare‐taxon recovery is a priority.

### 4.4 eDNA Sensitivity Calibration

By comparing eDNA metabarcoding with visual census in a controlled aquarium environment, we found that all taxa visually confirmed as present in the aquarium were also detected by eDNA. Nevertheless, a proportion of species detected by eDNA were not observed visually, thus potentially resulting in false positives. Such discrepancies may arise from undetected life stages (e.g., larvae), external contamination, PCR artifacts such as chimera formation, or taxonomic misassignments. Data curation strategies based on negative controls can influence eDNA metabarcoding results and introduce some degree of variability (Blanco et al. 2025, Takahashi et al. 2023, Sepulveda et al. 2019). Nevertheless, the use of *decontam* follows an approach adopted in the literature (Zhang 2023, Wesselmann et al. 2022, Maiello et al. 2024, Saltonstall et al. 2024, Saenz‐Agudelo et al. 2022) and provides a standardized and reproducible framework for contaminant detection. Furthermore, the inability to discriminate between two fish species sharing the same 12S fragment underscores a significant limitation of eDNA metabarcoding when targeting short regions, a necessity arising from the potentially degraded state of extracellular DNA. However, these ambiguous reads constituted a negligible proportion of the total, thereby suggesting that, contingent upon the objectives of the study, the quantitative information provided by read counts is indeed relevant (Salter et al. 2019; Rourke et al. 2022; Knudsen et al. 2019).

### 4.5 eDNA and Visual Censuses Comparison

In this single study, MiFish‐U eDNA metabarcoding enabled the evaluation of more than half (52%) of the fish species that have been recorded at least twice in Moorea since 2004. eDNA and visual censuses comparison revealed that eDNA metabarcoding successfully recovered the majority of taxa recorded visually. In addition, it also revealed several cryptic ( *Cirripectes quagga* , *Cirripectes variolosus* , *Nannosalarias nativitatis* , *Eviota albolineata* , *Callogobius sclateri* , *Pseudanthias mooreanus* ), rare, or transient species (*Gymnosarda unicolor*), a pattern that has been widely reported across temperate and tropical systems. (Boussarie et al. 2018; Polanco Fernández et al. 2021; West et al. 2020). The higher Shannon diversity in eDNA metabarcoding in comparison to visual surveys is consistent with the findings that eDNA metabarcoding integrates signals from elusive and nocturnal taxa, and sometimes from adjacent habitats, thereby broadening the detectable assemblage (Polanco Fernández et al. 2021; Valdivia‐Carrillo et al. 2021; Mathon et al. 2022). The significant, shared habitat and seasonal effects detected by both methods support the integration of eDNA metabarcoding with standardized visual programs to enhance coverage and trend detection (West et al. 2020).

### 4.6 Nanopore Sequencing Technology and Reads Processing

Despite the elevated error rate traditionally associated with Oxford Nanopore sequencing, the findings of this study demonstrate that contemporary Nanopore chemistry (R10.4.1) with high‐accuracy basecalling and consensus polishing generates reliable metabarcodes that are suitable for biodiversity monitoring as has been previously demonstrated (Baloğlu et al. 2021; Egeter et al. 2022; Maggini et al. 2024; Kasmi et al. 2024; Deremarque et al. 2025). The *Decona* pipeline, which has been extended, comprises quality and length filtering, clustering at 97% identity, alignment and polishing with minimap2–racon–medaka, and re‐clustering of consensuses—follows best practice and leverages recent pipelines tailored to long‐read data (Doorenspleet et al. 2025; Dubois et al. 2024; Li 2018). The accuracy of the assignment is contingent upon the comprehensive nature of the references provided. The MiFish fragment resolves the majority of fishes but leaves some congeners unresolved; locality‐specific enrichment and rigorous curation mitigate misassignments (Miya et al. 2015; Miya 2022). The regional database under consideration combines new Sanger sequences from Moorea vouchers with vetted GenBank records and trimmed primer regions. This approach follows the aforementioned recommendations and improves coverage for French Polynesia (Delrieu‐Trottin et al. 2019). This database currently constitutes the most extensive regional resource available for environmental DNA‐based monitoring of fish diversity and distribution in French Polynesia. This resource will serve as a critical tool for enhancing the accuracy of eDNA‐based monitoring. The success of the *Decona* pipeline, which combines sequence clustering and correction of consensus sequences, indicates that Nanopore's portability and real‐time capability make it a credible solution for in situ biodiversity monitoring—though it should be further benchmarked across more ecosystems and primer sets.

### 4.7 Temporal Resolution and Diel Dynamics

The results obtained demonstrate that biotic and environmental variables shape the spatial and temporal detectability of eDNA. eDNA metabarcoding provides a reliable snapshot of fish biodiversity that reflects circadian activity rhythms, thus underscoring its potential for temporally resolved ecological assessments in lagoon environments. As demonstrated in the relevant literature (Strickler et al. 2015; Barnes et al. 2014), marine studies indicate that persistence times are, as a general rule, shorter than in lakes. Furthermore, these studies demonstrate that these times are strongly modulated by UV exposure, temperature, and microbial activity. The enhanced detection of nocturnal species during the night—and reciprocal trends for diurnal species—support the hypothesis that fish activity modulates eDNA shedding, likely through increased movement, feeding, and metabolic activity. This underscores the temporal sensitivity of the method, while also highlighting its limitations: eDNA metabarcoding does not merely reflect biodiversity signals but rather serves as a dynamic indicator of organismal behavior at the time of sampling.

Whilst this temporal resolution is advantageous in terms of the detection of behavioral patterns, it can also act as a limitation with regard to the establishment of long‐term biodiversity baselines. In contrast to the methods based on sediment or biofilm, the analysis of eDNA in seawater appears to reflect ecological presence over brief time periods (hours rather than days), as degradation is accelerated by UV exposure, high temperatures, and microbial activity typical of tropical lagoons (Strickler et al. 2015; Tsuji et al. 2017; Pilliod et al. 2014; Barnes et al. 2014). This phenomenon was evidenced by the 24‐h sampling experiment, which demonstrated changes in community composition are observable across just a few hours. While this sensitivity is beneficial for detecting shifts in real time, it also suggests that temporal replication is essential to distinguish short‐term variability from long‐term trends. The results of this study therefore argue for time‐of‐day standardization when comparing sites or years and, when biological questions demand, explicit diel sampling to capture activity‐driven turnover (Jensen et al. 2022).

### 4.8 Spatial Signal and Hydrodynamic Transport

Spatially, we observed that some fish OTUs typically associated with outer reef slopes were detected inside lagoon samples. This phenomenon may result from hydrodynamic transport, particularly wave overtopping and tidal currents, which can carry eDNA across reef habitats. Empirical and modeling work indicates that although most marine eDNA signals are highly localized, transport across hundreds of meters to kilometers can occur depending on circulation, leading to partial spatial integration of communities (Tsuji et al. 2017; Closek et al. 2019). Such horizontal DNA movement offers the benefit of broader spatial integration, potentially increasing habitat coverage per sample. Yet, it also risks blurring spatial resolution, as signals may not reflect the local presence of organisms. Fine‐scale habitat structure is nonetheless resolvable at tens of meters (West et al. 2020), which matches the strong habitat partitioning we observed. Consequently, survey design should incorporate local hydrodynamics, such as the position of the sampling relative to the direction of sea currents, the reef crest, and the prevailing currents, when interpreting cross‐habitat detections. This underscores a fundamental tension in eDNA metabarcoding methods: sensitivity vs. spatial imprecision.

## Author Contributions

**Lucie Cartairade:** conceptualization (equal), data curation (lead), formal analysis (lead), methodology (equal), visualization (lead), writing – original draft (lead), writing – review and editing (equal). **Julie Poulain:** conceptualization (equal), methodology (equal), validation (equal), writing – review and editing (equal). **Quentin Carradec:** methodology (equal), validation (equal), writing – review and editing (equal). **Sophie Mangenot:** data curation (equal), methodology (equal), validation (equal). **Patrick Wincker:** conceptualization (equal), funding acquisition (equal), methodology (equal), supervision (equal), validation (equal). **Serge Planes:** conceptualization (equal), funding acquisition (equal), methodology (equal), supervision (equal), validation (equal), writing – original draft (supporting), writing – review and editing (equal).

## Funding

This research was financially supported by the Direction de l'Environnement (DIREN), Marepolis, the ANRT, Genoscope, the Commissariat à l'Energie Atomique et aux Energies Alternatives (CEA), and France Génomique (ANR‐10‐INBS‐09), whose contributions were instrumental in enabling both the field operations and the analytical developments undertaken in this study.

## Conflicts of Interest

The authors declare no conflicts of interest.

## Supporting information


**Figure S1:** ece373254‐sup‐0001‐FigureS1.jpg.

**Figure S2:** ece373254‐sup‐0002‐FigureS2.jpg.

**Figure S3:** ece373254‐sup‐0003‐FigureS3.jpg.

## Data Availability

The whole pipeline used is described in the public git repository: https://github.com/LucieCartairade/eDNA_Methodology. The curated reference database for the 12S gene portion of French Polynesia fish is available at the following link: https://zenodo.org/records/16969829. FASTQ reads are submitted in SRA, with the PRJNA1418494 accession number.
