# Supplementary figures and images for: Optimizing a Novel eDNA‐Based Framework for Reef Fish Biodiversity Monitoring Using an Autonomous Filtration System and in situ Nanopore Sequencing
Source: Ecol Evol. 2026 Mar 23;16(3):e73254. doi: 10.1002/ece3.73254 (PMC13093698; doi:10.1002/ece3.73254)

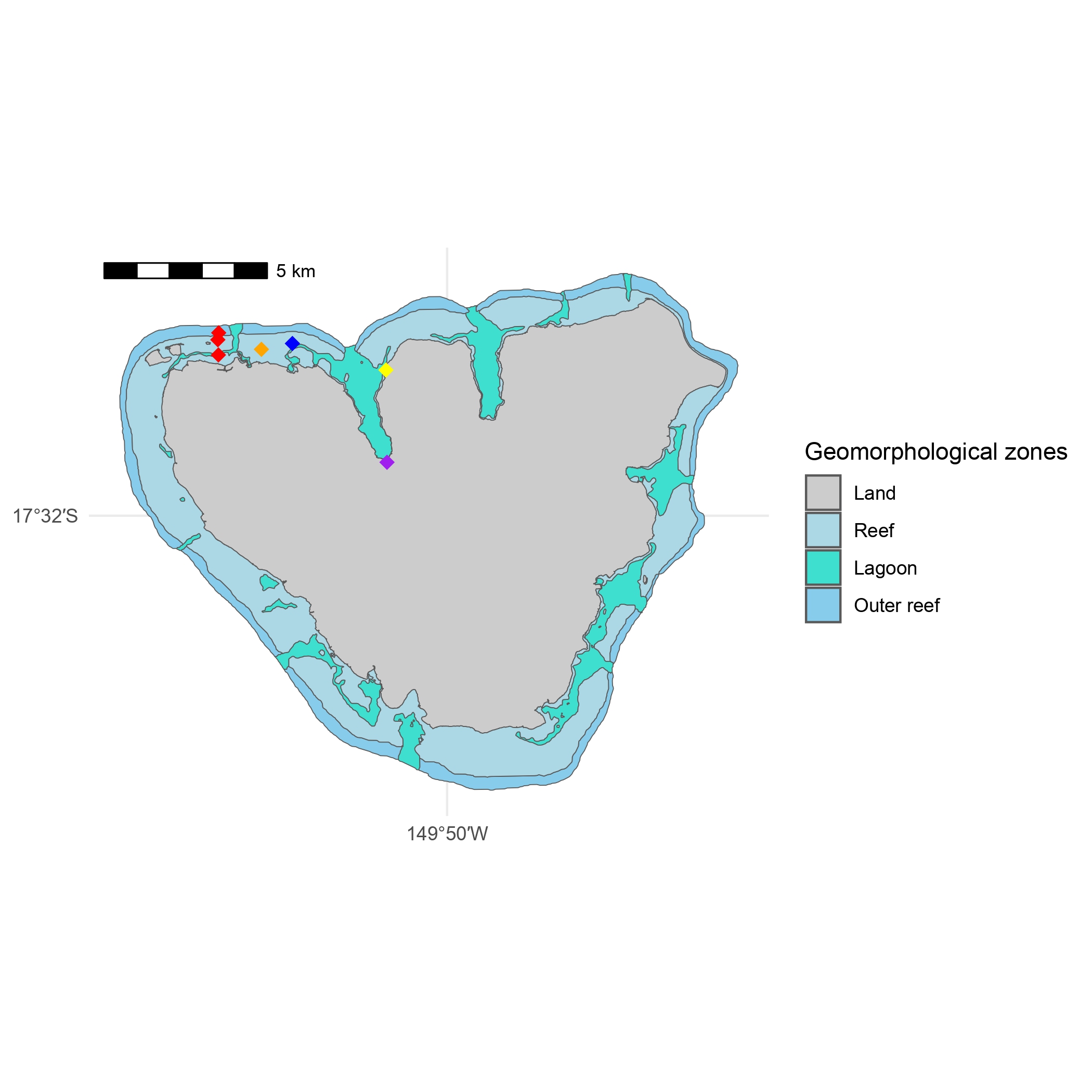

Supplement: Supplementary file 1 — Figure S1: ece373254‐sup‐0001‐FigureS1.jpg. [file ECE3-16-e73254-s002.jpg]

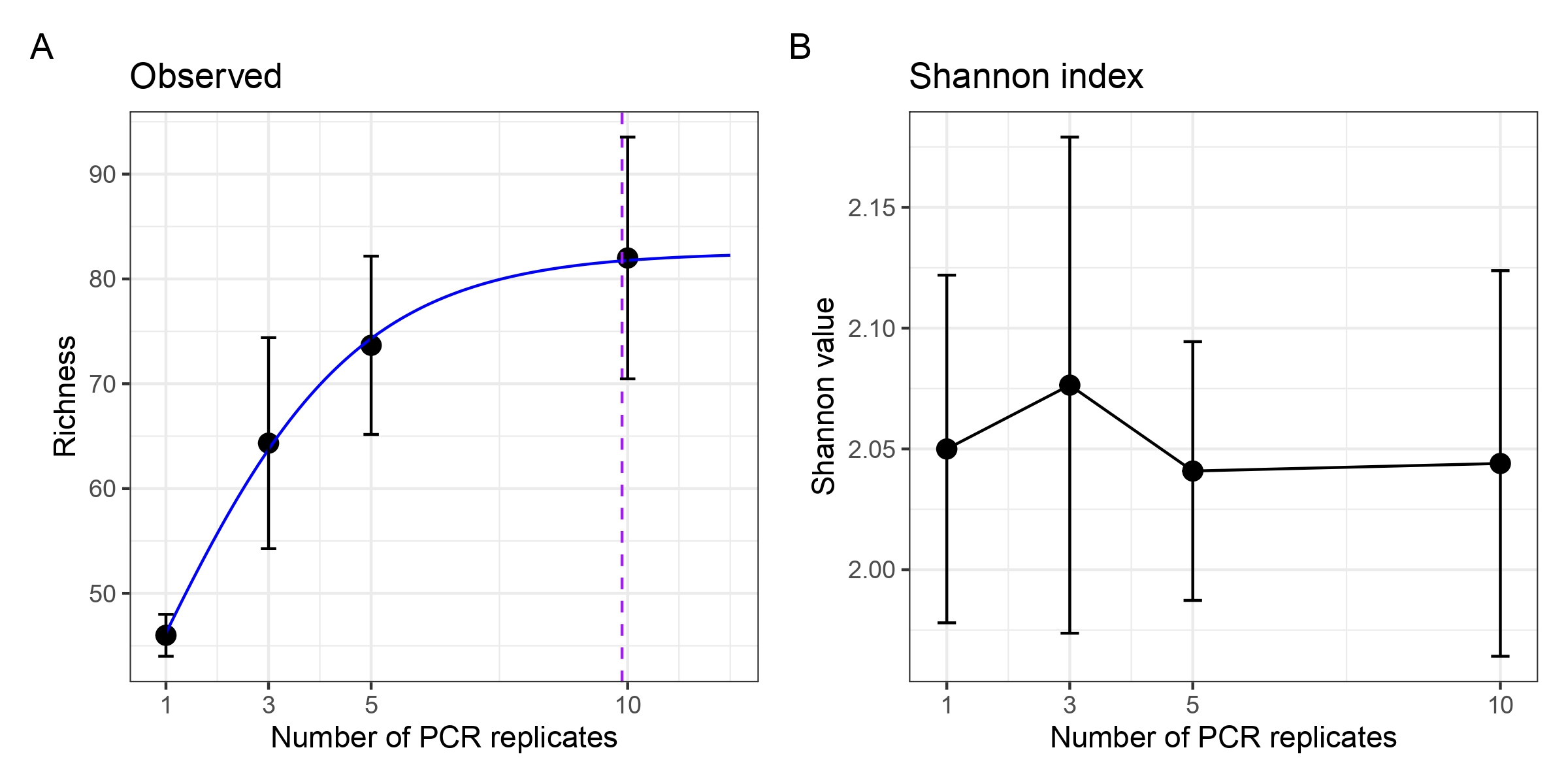

Supplement: Supplementary file 2 — Figure S2: ece373254‐sup‐0002‐FigureS2.jpg. [file ECE3-16-e73254-s001.jpg]

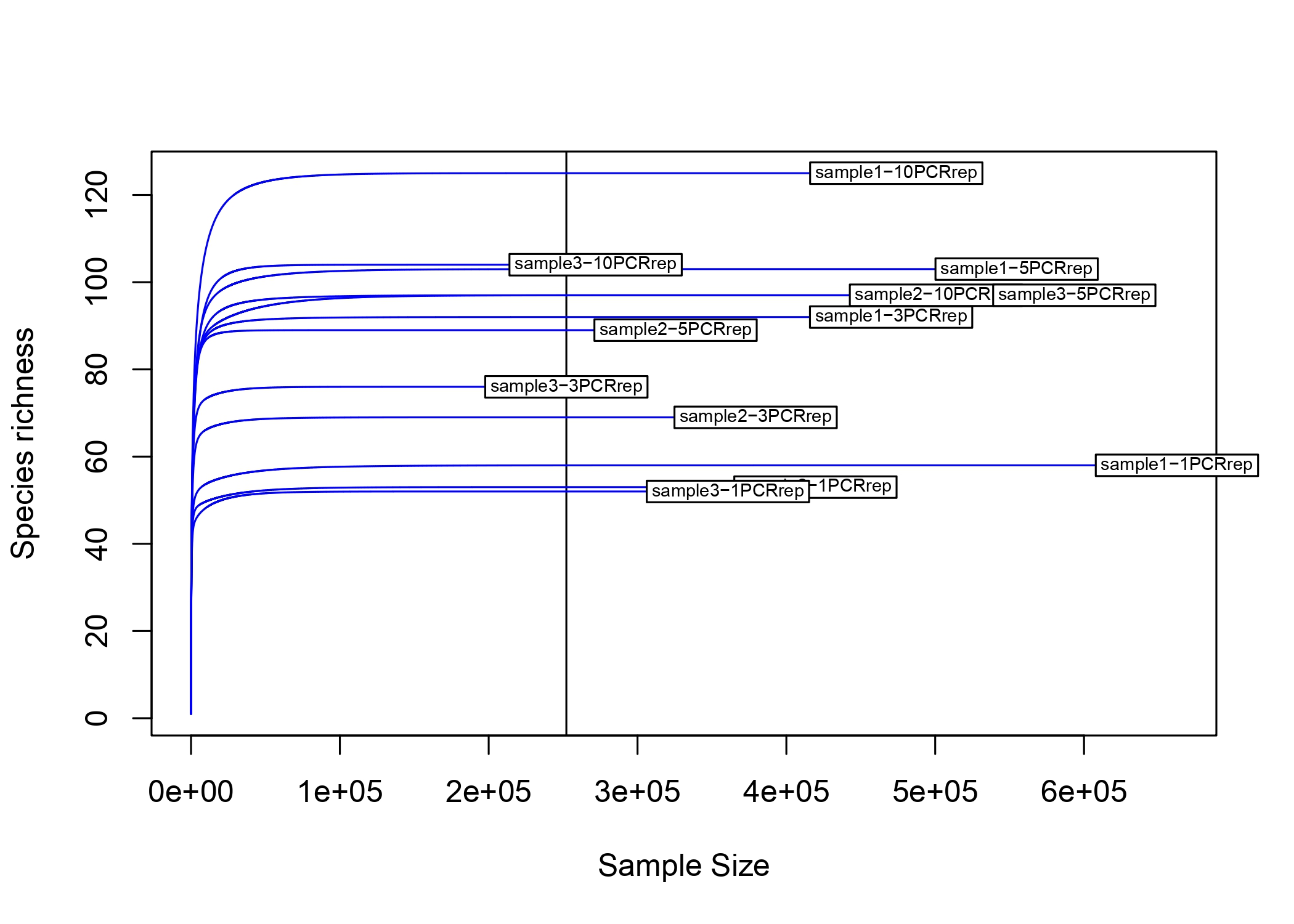

Supplement: Supplementary file 3 — Figure S3: ece373254‐sup‐0003‐FigureS3.jpg. [file ECE3-16-e73254-s003.jpg]
